# Clinical Implementation of Wearable-Derived Sleep and Activity Reporting for Inpatient Psychiatric Monitoring

**DOI:** 10.2196/88466

**Published:** 2026-09-21

**Authors:** Brien William Culhane, Robert Dennis Patterson, Habiballah Rahimi-Eichi, Agustin Go Yip, Kayla Huesman, Kristin Kostick-Quenet, Joshua Salvi, Philippe Beauchamp, Kerry James Ressler, Justin Taylor Baker

**Affiliations:** 1 Institute for Technology in Psychiatry, McLean Hospital, 115 Mill Street, Belmont, MA, 02478, United States, 1 617-855-3913; 2 Department of Psychiatry, Harvard Medical School, Boston, MA, United States; 3 Center for Medical Ethics and Health Policy, Baylor College of Medicine, Houston, TX, United States; 4 Department of Psychiatry, McGill University, Quebec City, QC, Canada

**Keywords:** implementation, behavioral measurement, inpatient, psychiatric, actigraphy, sleep, digital health technology, wearable

## Abstract

**Background:**

Sleep is a core component of psychiatric assessment, yet inpatient monitoring typically relies on brief observational checks that are subjective, variable, and sometimes disruptive. Wearable devices offer a means of capturing continuous, objective sleep and activity data without disturbing patients. Although digital health technologies are increasingly used in psychiatric research, little is known about how wearable-derived data can be integrated into routine inpatient workflows or used meaningfully by clinicians.

**Objective:**

This implementation aimed to evaluate the feasibility, usability, and workflow integration of a wearable-derived sleep and activity reporting system within an adult psychiatric inpatient unit.

**Methods:**

The implementation unfolded in 2 phases at a single 21-bed adult inpatient unit at a psychiatric hospital in Massachusetts. Patients were offered a wrist-worn GENEActiv actigraphy device upon admission. Raw accelerometry data were processed using the DPSleep pipeline to derive daily sleep and activity metrics for patients participating in the implementation. Sleep and activity reports combining graphical summaries and natural language summaries of sleep, activity, and medication data were iteratively refined and delivered to psychiatrists providing patient care. Semistructured qualitative interviews were conducted with clinicians and unit staff to gather feedback on the sleep and activity report prototype and discuss barriers to and facilitators of implementation. Interview data were coded and analyzed by a team of 2.

**Implementation (Results):**

During phase 1 of the implementation, 155 patients were admitted, of whom 88 (56.8%) were offered a device and 68 (77.3%) accepted it. Sleep and activity reports were generated for 61.8% (42/68) of patients wearing a device during this phase. During phase 2 of the implementation, automation reduced report generation time from approximately 5 days to under 24 hours. Only 1 of the 3 psychiatrists on the unit regularly used the reports in routine care. Reports were most useful for reconciling discrepancies between patient and nursing sleep estimates and for supporting clinical conversations about sleep patterns and medication adherence between clinician and patient. Clinicians who had not yet used the reports expressed conceptual interest but emphasized the need for integration in the electronic medical record, reliably available “last-night” sleep data, and simplified design. Barriers included challenges in the speed, reliability, and clarity of the data; variable staff buy-in; and disconnects between the research and clinical teams running the implementation.

**Conclusions:**

This implementation suggests that wearable-derived sleep and activity data reporting is technically feasible in inpatient psychiatry. This data reporting potentially offers clinically meaningful insights. Use of the reports was concentrated in 1 of the 3 psychiatrists on the unit, who served as an early adopter and project champion (AGY). Sustainable use and broad clinical uptake are more likely with reliable, near-instantaneous data transfer; electronic medical record integration; and shared implementation ownership across staff levels.

## Introduction

### Context

Sleep is regularly assessed in inpatient care as a core symptom of psychiatric illness [1]. Standard overnight checks rely on staff observation and are inherently subjective, sometimes disrupting patient sleep [2]. Wearable devices offer a means of obtaining continuous, objective sleep and activity data without disturbing patients, presenting an opportunity to enhance the accuracy and efficiency of inpatient monitoring [3].

### Problem Statement

This implementation aimed to address a health system challenge identified by the World Health Organization—specifically, the lack of reliable, high-quality data to support clinical decision-making (challenge 1.3) [4]. Although many have advocated for integrating digital data into psychiatric practice [5-7], most prior efforts have centered on feasibility testing or predictive modeling rather than sustained clinical integration. Consequently, little is known about how wearable-derived data can be incorporated into existing workflows or meaningfully used by clinicians within inpatient psychiatric care.

### Similar Interventions

Previous research illustrates both the promise and challenges of using digital measures in hospital settings. Kooij et al [8] examined the use of wireless wearable sensors on teaching hospital wards and reported that nurses perceived benefits in the early detection of physiological deterioration among hospitalized patients. Similarly, Pickham et al [9] conducted a pragmatic randomized controlled trial evaluating wearable patient sensors for enhancing nursing care delivery. In psychiatry, Hong et al [10] used wearable-derived modeling to forecast symptom trajectories, whereas Odachi et al [11] and Durrani et al [12] evaluated the feasibility of feeding back sleep and activity data to clinicians and patients. While these studies have examined the feasibility of wearable-derived data use among clinicians within a research framework, none have attempted to integrate systems for wearable-derived data collection and delivery in clinical care through an implementation framework. By “implementation framework,” we refer to a structured, iterative process of adapting device management, data processing, and data delivery procedures based on clinician feedback with the goal of embedding the system into existing inpatient workflows. This approach extends beyond feasibility testing to understanding the organizational, usability, and workflow determinants of sustained adoption. This approach answers the call in the study by Odachi et al [11] for the development of guidelines for implementation of digital health tools through collaboration among researchers, engineers, and clinical staff—especially exploring how to incorporate data into routine care.

### Aims and Objectives

Our implementation was a collaboration between a research laboratory and clinical staff at a single adult inpatient unit at a major psychiatric hospital (Table 1). We sought to explore and evaluate methods for integrating wearable-derived sleep and activity feedback into psychiatric inpatient workflows through a clinician-partnered implementation. Using an iterative design framework within routine inpatient psychiatric care, we adapted project roles, data feedback formats, and delivery mechanisms throughout the implementation in response to feedback from participating clinical staff. The primary aim of this implementation was to evaluate how objective sleep and activity data could be effectively collected, reported, and incorporated into clinical decision-making within a psychiatric inpatient setting. Specifically, we sought to develop, deploy, and iteratively refine a prototypical sleep and activity report for clinician use, assessing its feasibility, usability, and integration into clinical workflow.

**Table 1.** Implementation setting characteristics.

Characteristics	Values
Unit size	21-bed locked unit
Length of stay (days), mean (SD)	17 (23)
Patient age range (years)	18‐65
Patient sex (n=181), n (%)^a^	
Female	66 (36.5)
Male	106 (58.6)
Unreported	9 (5.0)
Race (White),	145 (80)
Payer (Medicaid and/or Medicare), n	17
Common diagnoses	Major depressive disorder, schizophrenia spectrum, bipolar disorder, and borderline personality disorder

a Some patients did not report sex, so percentages do not add up to 100%.

## Methods

### Blueprint Summary

**Figure 1.**
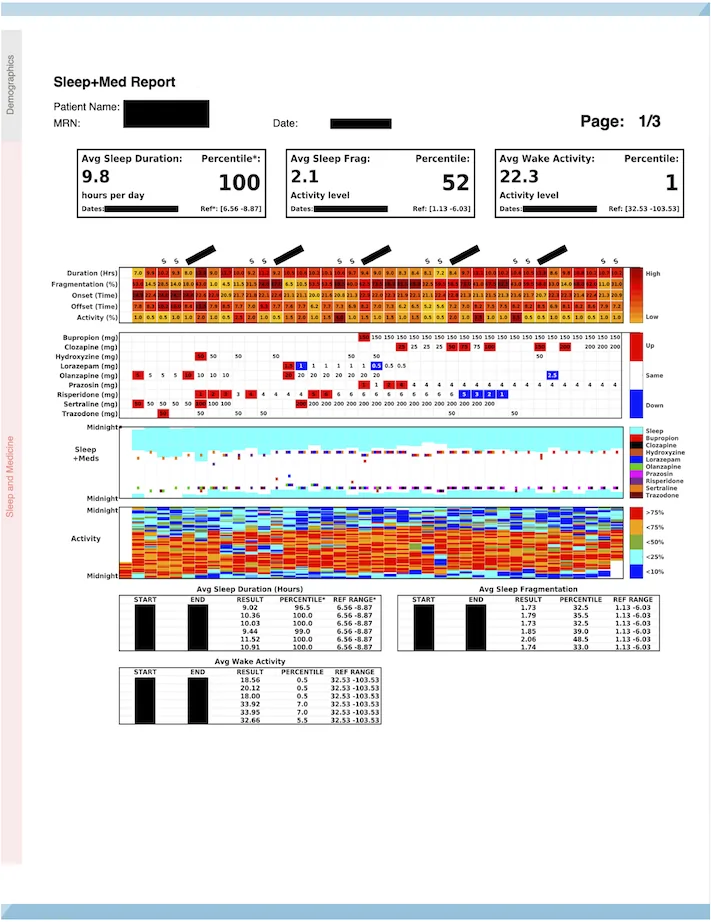
Graphical sleep and activity report.

**Figure 2.**
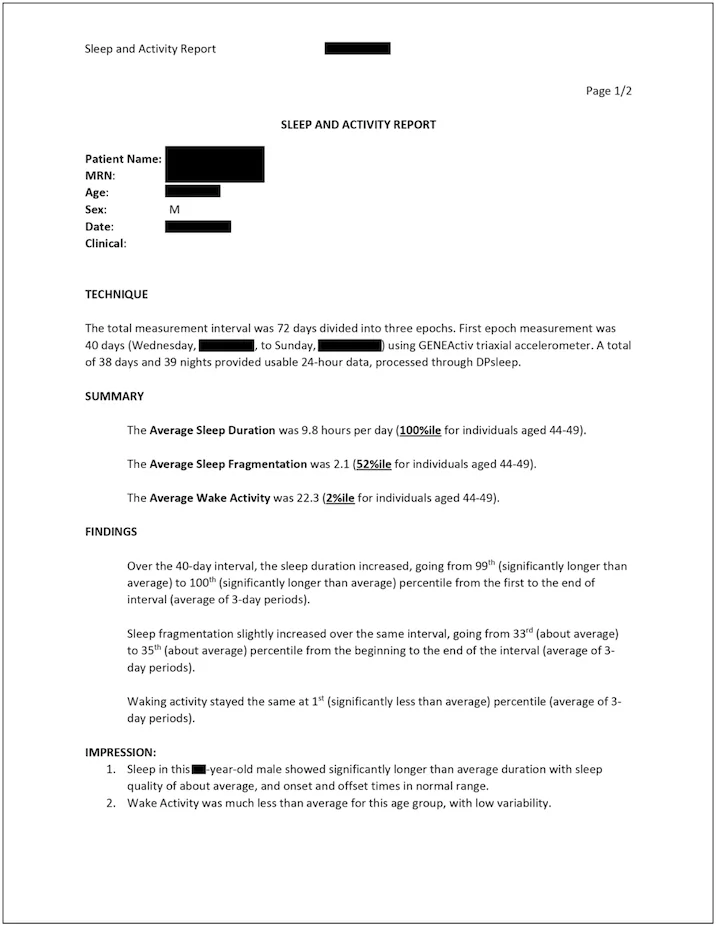
Natural language summary of the graphical sleep and activity report.

Implementation unfolded in 2 phases (Table 2; Multimedia Appendix 1). Phase 1 (August 2023-December 2023) tested several methods of device management, report request, and report delivery. During this phase, we began writing natural language summaries of the graphical report (Figure 1) in response to challenges of interpretability (Figure 2). Uptake metrics were tracked throughout phase 1, including number of admissions, devices offered to patients, devices accepted by patients, and reports generated. Phase 2 (January 2024-June 2024) established an email-based request model for reports and streamlined data turnaround times, fully automating the generation of graphical reports and natural language summaries. In this phase, project resources were shifted away from driving uptake on the unit and rededicated to streamlining systems for report generation, report delivery, and gathering clinician feedback. This shift was in response to low uptake in phase 1 and served as an opportunity to clarify and address the challenges identified in phase 1. Consequently, uptake metrics were not tracked, and reports were only generated at clinician request.

**Table 2.** Evolution of procedures for device distribution, report generation, and report delivery (August 2023-June 2024).

	Phase 1				Phase 2 (January 2024-June 2024)
	August 2023-September 2023	October 2023	November 2023	December 2023	
Enrollment	Nurse offers patient a device at admission	No change	No change	Nurse offers patient a device at admission	Nurse offers patient a device at admission
Recording period	Devices are worn until a report is requested via REDCap survey	*Devices are worn until weekly device collection on the following Thursday* ^a^	No change	Devices are worn until weekly device collection on the following Thursday	*Devices are worn until a report is requested via email*
Watch collection	The project RA^b^ collects the patient’s device within 24 h of a report request; data for the requested report are uploaded by end of day	*Once a week, the project RA collects all devices worn for ≥3 d; data for all participating patients are uploaded by end of day*	No change	Once a week, the project RA collects all devices worn for ≥3 d; data for all participating patients are uploaded by end of day	*The project RA collects the patient’s device within 24 h of a report request; data for the requested report are uploaded by end of day*
Report generation	Automated in-house software produces a graphical report	*Automated in-house software produces graphical reports; text summaries for all graphical reports are handwritten on Sunday by laboratory staff*	*Automated in-house software produces graphical reports; text summaries for all graphical reports are semiautomated with handwritten details and done on Sunday by laboratory staff*	Automated in-house software produces graphical reports; text summaries for all graphical reports are semiautomated with handwritten details and done on Sunday by laboratory staff	*Automated in-house software produces a graphical report; text summaries for all graphical reports are fully automated*
Report delivery	Report emailed to requesting clinical staff member within 48 h of request	Reports brought to rounds and presented by the project RA on Tuesdays (5 d after device collection)	*The project RA gives reports to nurses and highlights key information for each patient on Tuesday morning before rounds (5 d after device collection)*	The project RA gives reports to nurses and highlights key information for each patient on Tuesday morning before rounds	*Reports are emailed or sent via secure file transfer to clinical staff within 48 h of request and sometimes as soon as within 3 h of request*

a Italics indicate procedural changes introduced at each time point relative to the prior period.

b RA: research assistant.

At the conclusion of phase 2, semistructured interviews were conducted with a convenience sample of 8 psychiatrists from across the hospital to explore their impressions of the sleep and activity report. Of these psychiatrists, only 1 had used the sleep and activity report in practice. This was the unit medical director (AGY), who is listed as an author of this paper and who participated in the implementation as a highly engaged early adopter and project champion on the unit. Additionally, the unit medical (AGY) and nursing (KH) directors were each interviewed separately to discuss the course of the implementation on the unit.

Interviews were analyzed using the rigorous and accelerated data reduction technique developed by Watkins [13]. Two members of the research team (BWC and RDP) collaboratively developed a “coding scheme” organizing the interview data into categories and then independently coded the interview transcripts and met regularly to engage in consensus-reaching discussions to resolve any discrepancies in coding. Codes were then grouped into overarching themes comprising feedback on the report, workflow integration, and barriers to and facilitators of implementation.

### Technical Design

Sleep and activity were monitored using waterproof, wrist-worn GENEActiv devices (Activinsights Ltd). We chose this device for its capacity to collect continuous, raw triaxial accelerometer data at a fixed rate without frequent recharging. Devices sampled at 20 Hz for up to 30 days.

Data were processed using DPSleep, an open-source longitudinal pipeline for sleep analysis from accelerometer data [14]. Extracted sleep parameters included sleep onset time, wake time, sleep duration, and sleep fragmentation. Fragmentation index was calculated by the slightly modified percentage of mobile minutes during the sleep epoch as described in the study by Rahimi-Eichi et al [15] and is intended as a proxy measure for sleep quality. Minute-level activity was derived and normalized within participants expressed as relative activity percentiles.

Medication administration data were retrieved from the electronic medical record (EMR) via the institution’s data warehouse. The graphical section of the report was generated in MATLAB 2019b (MathWorks, Inc). The natural language section was generated using a Python script that summarized key data from the graphical report using simple heuristics; large language models were not used in data processing or report generation.

### Target

Our implementation intended to support inpatient psychiatrists in clinical decision-making by reporting objective inpatient sleep and activity. In the implementation, we focused on discerning how psychiatrists wished to see data presented in the report and how best to integrate reports into psychiatrists’ workflow.

### Ethical Considerations

All protected health information was stored on encrypted institutional servers and HIPAA (Health Insurance Portability and Accountability Act)-compliant cloud storage. Data collection and management followed institutional governance procedures.

Patient participation was voluntary, and consent to wear a device was obtained verbally as part of routine clinical workflow consistent with institutional review board determination that the project qualified as an implementation initiative rather than human subject research. This designation as not human subject research covered both patient participation and the collection of feedback from clinical staff via semistructured interviews.

### Interoperability

The implementation architecture comprised three integrated modules: (1) data acquisition using GENEActiv devices and software, (2) processing and analysis using DPSleep, and (3) report generation (MATLAB and Python) and delivery (email and secure file transfer).

### Participating Entities

The implementation was conducted at McLean Hospital, a division of Mass General Brigham and an affiliate of Harvard Medical School. The implementation was coordinated by the McLean Institute for Technology in Psychiatry and Functional Neuroimaging and Bioinformatics Laboratory in collaboration with unit leadership. All intellectual property is owned by Mass General Brigham.

### Budget Planning

The implementation was funded by discretionary institutional resources from McLean Institute for Technology in Psychiatry. The implementation period covered August 2023 to June 2024, with total expenditures confined to existing institutional budgets; no additional financial resources were required beyond routine operational support. No external grant funding was used.

Hardware costs consisted of GENEActiv Original devices (approximately US $275 per device), which were supplied from an existing inventory in the Functional Neuroimaging and Bioinformatics Laboratory purchased for use in prior studies via grants and discretionary funds. Thirty devices were allocated for this implementation. Data analysis and reporting relied exclusively on in-house–developed software. Personnel time—including a single research assistant managing device turnover and data processing—represented most ongoing costs.

### Sustainability

Sustainability was assessed in terms of patient participation, technical feasibility, and potential institutionalization. Patient willingness to wear devices was high, and nurses reliably retrieved devices before discharge, demonstrating operational feasibility. One patient inadvertently took a device home upon discharge and promptly mailed it back; no watches were destroyed. Automation of report generation markedly reduced personnel burden, increasing scalability.

Long-term sustainability will depend on (1) embedding automated data transfer and report visualization within the EMR to ensure rapid and reliable access, (2) institutional recognition of objective sleep reporting as a quality of care metric, and (3) sustained cross-departmental engagement fostered by a shared sense of ownership among all participating staff.

Financially, the implementation could be maintained with minimal additional cost once incorporated into standard clinical workflows. Strategically, this initiative aligns with the hospital’s and the World Health Organization’s digital health objectives promoting interoperable, data-driven clinical decision support.

## Implementation (Results)

### Coverage

The implementation was conducted at a single site—a 21-bed inpatient unit within a 238-bed psychiatric hospital in Massachusetts, United States. The initiative represents a subnational implementation focused on feasibility and workflow adaptation prior to potential expansion locally and beyond.

During phase 1 of the implementation (August 2023 to December 2023), 155 patients were admitted to the unit, of whom 88 (56.8%) were offered a device and 68 (77.3%) accepted it. During this phase, 61.8% (42/68) of patients wearing a device had at least one sleep and activity report generated and delivered to their psychiatrist. Reports were not generated in all cases of device wear because report generation in this phase began with a request model and clinicians did not request reports for all participating patients (Table 2).

During phase 2 (January 2024 to June 2024), complete automation of report generation markedly improved timeliness: average report generation time decreased from approximately 5 days to less than 24 hours, and in several instances, same-day delivery was achieved. During this phase, 34 reports were generated and delivered, of which 32 (94.1%) were requested by the medical director (AGY) on the unit. One of the psychiatrists present in phase 1 had left the unit by this point, and a team of rotating clinicians covered this psychiatrist’s role on the unit for the duration of phase 2. Two reports were requested by rotating clinicians. No reports were requested by the third psychiatrist on the unit.

### Outcomes

#### Operational Outcomes

Implementation resulted in secure, reliable data capture and automated generation of clinician-readable sleep and activity summaries. Integration of medication data from the EMR was technically feasible. Patients demonstrated willingness to wear devices for the duration of inpatient stay.

#### Clinical Uptake

Of the 3 psychiatrists on the unit, only 1—the medical director (AGY)—used the reports as part of routine clinical practice. This clinician served as a project champion and coleader of the implementation as well as the primary real-world user. The 2 other psychiatrists on the unit were aware of the implementation but did not regularly engage with reports. One requested a single report early in the implementation. Both received information from the reports in phase 2 when we delivered key information in clinical rounds, but neither participated beyond this.

The medical director (AGY) most often used reports to reconcile discrepancies between nursing documentation and patient self-reports of sleep. In these cases, the objective data provided a third reference point beyond the patient’s impression and the nurse’s sleep report. The medical director (AGY) described this as an opportunity for therapeutic dialogue with patients and a means of grounding sleep discussions in objective evidence. On at least 2 occasions, the medical director (AGY) used sleep duration and quality measures to evaluate a recent change in sleep medication (sedative-hypnotic) and shared the data with the patient, which the medical director (AGY) felt reinforced the clinical utility of the medication change. Visualizations of daily sleep duration were particularly useful to the medical director (AGY) for assessing stability or change, whereas summary metrics averaged throughout the stay were less actionable given that treatment decisions regarding sleep are often made based on duration data from the night prior or a few nights prior. Fragmentation data (a proxy measure for sleep quality) were seen as potentially valuable but required clearer interpretive framing. The addition of the natural language summary improved the report’s readability, but it was not thought to be particularly more clinically actionable by the medical director (AGY), who cited the timeliness and reliability of the report as the greatest barriers to clinical use.

#### Clinician Impressions From Prototype Review

In addition to the medical director (AGY), a convenience sample of 7 psychiatrists from across the hospital who had not participated in the implementation was interviewed to gather broader perspectives on report design and potential clinical use. These clinicians reviewed the final report prototype but did not use it in practice. Their feedback reflected support for the concept but uncertainty regarding its practical application without direct integration into the EMR. Most found the daily plots of key sleep metrics intuitive and most useful. Reviews of the medication sections were mixed; some psychiatrists thought these sections were redundant, whereas others found them a useful tool for illustrating effects of medication adherence to patients. Reviews of the natural language summaries were similarly mixed: while some psychiatrists were indifferent about its potential utility, others were enthusiastic about the potential usefulness of a written summary for interpreting the report and identifying key actionable metrics and clinical impressions of the sleep and activity data. Several expressed that the report would be most useful if it displayed key sleep metrics (eg, duration and fragmentation) from the night prior in a concise table integrated into the EMR before morning rounds, with the option to engage with more granular data (eg, duration for each day in the previous week) as desired.

#### Patient and Staff Experience

Patients were generally receptive to wearing devices (68/88, 77.3% of admits who were offered a device accepted), with 20 recorded reasons for refusals—5 (25%) of which were due to privacy or surveillance concerns, and the rest were due to general unwillingness to participate without particular reason. Nursing staff offered and collected devices fairly reliably, although their consistency and speed seemed to depend on psychiatrist engagement and the perceived clinical importance of the implementation according to the views of the medical (AGY) and nursing (KH) directors. Some patients expressed interest in seeing their sleep data, which was possible through discussion of a sleep and activity report with their psychiatrist.

### Lessons Learned

#### Guiding Principles for Design

Across interviews reviewing the report prototype, several design principles emerged. To maximize their utility, reports should appear within the EMR before morning rounds. They should present key sleep metrics for the prior night, with more granular data available as needed. Clinicians favored daily-level information and intuitive visualization over aggregated metrics. Clarity and cognitive burden were key factors. Most clinicians reported that they would spend roughly 15 to 30 seconds per patient on average reviewing the report.

#### Barriers and Facilitators

Implementation success was shaped primarily by technical and interpersonal factors. Engagement by the medical director (AGY) strongly influenced unit buy-in, whereas limited enthusiasm among other psychiatrists reduced unit buy-in overall. Given that nurses were asked to offer devices to patients at admission, challenges inspiring buy-in among the nursing staff significantly affected the number of patients wearing a device. This was particularly true among nursing staff who perceived the additional responsibilities of device distribution and tracking as burdensome and of questionable value—especially as 2 of the 3 psychiatrists on the unit were not actively interested in the reports. Technical barriers to the reliability and speed of report delivery further constrained adoption.

A broader barrier to implementation was the perceived divide between clinical care and the implementation, often considered purely “research” by unit staff. Many staff members referred to the implementation initiative as “the sleep study,” reflecting a persistent belief that wearable use was “research” and not “clinical” practice. This perception was reinforced by unfamiliar quantitative language (eg, percentiles and fragmentation scores) that did not match the narrative language that unit staff typically use to discuss sleep (eg, “Did they sleep okay?” or “She said she wasn’t sleeping very well”). This mismatch further inhibited report implementation and exacerbated the sense among nursing staff that they were being asked to perform a “research” activity in providing and tracking devices.

Patients were broadly willing to wear the device, although in a few cases, patients reported discomfort in wearing it or wearing it alongside their personal watches, especially given that patients must also wear a hospital bracelet during their stay.

#### Success Factors and Sustainability

Despite these challenges, the implementation achieved high patient participation and full automation of report generation by its conclusion. No adverse events were reported. Some patients showed significant interest in seeing their sleep and activity data. The medical director (AGY) regularly used the reports throughout the initiative and considered them useful when promptly available. The leadership and persistence of the medical (AGY) and nursing (KH) directors were critical facilitators of the implementation.

Overall, the results indicate that wearable-derived sleep and activity reporting can be feasibly implemented within inpatient psychiatry. Sustainability depends on data timeliness, EMR integration, multilevel ownership, and a shared understanding of the clinical purpose of the data (Table 3).

**Table 3.** Key takeaways for future implementations of inpatient sleep and activity reporting.

Area	Takeaways
Implementation design	Engage representatives from all staff levels early and continuously to achieve a sense of shared ownership and bilateral feedback. Recruit “project champions” among each level of staff involved in the implementation. Provide education alongside implementation of digital health tools. Employ dedicated project staff focused on the implementation. Educate and encourage clinicians in terms of discussing sleep and activity data with patients. Make sleep and activity data available to patients and encourage discussion with clinicians.
Technical design	Integrate sleep and activity data reporting into the electronic medical record. Display concise “last-night” sleep data (eg, duration, fragmentation, onset, and offset) each morning before rounds. Provide options for clinicians to engage with more granular data as desired (eg, duration by day across the previous week). Prioritize delivering basic metrics quickly, currently, and reliably—unreliability quickly leads to low buy-in and low user engagement. Use devices that allow for wireless, near-instantaneous data processing, quality control, and delivery. Ensure that processing systems are accurate enough so as not to require human checking prior to data delivery. When possible, use diagnostic-specific population data for data norming.
Evaluation	Study clinician and patient behavior change in relation to data provision. Determine which metrics have the greatest impact on treatment and behavior change. Further assess clinician and patient attitudes toward wearable-derived data provision.

## Discussion

### Principal Findings

This implementation explored methods of collecting and reporting objective, wearable-derived sleep and activity data within inpatient psychiatric care. Most patients (68/88, 77.3% of admits who were offered a device) were willing to wear the device. Reports were particularly useful for reconciling discrepancies between patient and nursing reports of sleep, offering an additional, objective reference point to support clinical decision-making. However, uptake among psychiatrists was limited, with sustained use only by the medical director (AGY) who co-led the initiative. Therefore, the demonstrated clinical utility of the report was concentrated in a highly engaged early adopter and may not yet reflect routine uptake among average end users. Uptake of report use was primarily limited due to issues of clarity, lack of EMR integration, speed and reliability of report delivery, and limited buy-in from clinical staff.

These findings echo earlier reports that highlight both the promise and difficulty of integrating digital measures into clinical workflows. Kooij et al [8] and Pickham et al [9] similarly found that, while wearable systems can enhance early detection and clinical awareness, sustained use depends on how seamlessly data fit into existing work routines. Similarly, Odachi et al [11] and Durrani et al [12] observed clinician interest in sleep feedback but noted practical barriers related to usability and workflow integration.

Our findings complement those of prior work by moving beyond feasibility and prediction studies to an implementation of wearable-derived sleep and activity feedback within psychiatric inpatient care using an iterative design framework that evolved procedures in response to clinician feedback and operational constraints. This approach allowed us to refine multiple methods for device management, data processing, and report delivery, yielding a more comprehensive understanding of the technical and interpersonal factors that shape feasibility in real-world inpatient psychiatric contexts.

Our implementation aligns with calls from Mohr et al [5], Westheimer et al [6], and Oudin et al [7] to move beyond feasibility demonstrations toward pragmatic integration of digital data into psychiatric decision-making. Specifically, clinicians’ preference for simply displayed data that appear directly in the EMR prior to rounds reinforces that usability and timing are critical.

Our experience underscores the need to address both technical and interpersonal infrastructure in digital health implementation in accordance with previous inpatient monitoring studies [8,9]. Achieving buy-in at all levels of staff participating in the implementation is crucial. To this end, we recommend recruiting “project champions” at all levels and keeping in communication with them throughout the implementation. Once buy-in and compliance are achieved, it is crucial that technical infrastructure be able to reliably meet the demands and expectations of users. Our system’s reliance on software in-development and nonnetworked data collection limited report timeliness. The lack of near–real-time data transfer constrained the immediacy that clinicians require in fast-paced inpatient settings. These limitations are consistent with prior observations that technical latency and data opacity undermine clinical confidence in wearable-derived data [10,12].

Successes and indications of feasibility were also evident. Most patients (68/88, 77.3% of admits who were offered a device) were willing to wear it for the duration of their stay. The reports were used by 1 of the 3 psychiatrists on the unit, who also participated in this implementation as a key member. He found the reports useful—both as a tool for insight into patient sleep and activity and for discussing medication and sleep with patients.

### Limitations

Our implementation was conducted on a single unit and is therefore limited in its generalizability. Our capacity for interviewing was limited, so our interviews reviewing the implementation primarily included the medical (AGY) and nursing (KH) directors on the unit—both of whom are authors of this paper—introducing potential bias in the perspectives we gathered regarding the barriers and facilitators encountered by the implementation, as well as the suggestions for further implementation initiatives. Additionally, much of the feedback we received on the utility and design of our reports was derived from prototype review with psychiatrists not directly involved in the implementation rather than real-world users. These psychiatrists were selected by the implementation team and are thereby subject to potential sampling bias. Finally, conducting this implementation using technologies in development limits our ability to assess the feasibility of implementing commercial-grade wearable-derived sleep and activity reporting on inpatient units.

### Future Recommendations

Future implementations should pursue both technical optimization and organizational integration. Technically, establishing real-time, automated data pipelines and embedding outputs directly into EMR interfaces would improve timeliness and accessibility. Organizationally, fostering multidisciplinary ownership—through sustained involvement of all staff levels, regular feedback, and identification of “project champions”—could enhance buy-in and sustainability.

Future research should more closely assess the clinical impact of such tools on treatment outcomes, clinician behavior change, and patient engagement. Evaluation of how objective sleep and activity data influence diagnostic decisions, medication adjustments, and patient satisfaction will be essential to justify broader adoption. Finally, educational initiatives to familiarize clinicians with the interpretation of wearable-derived metrics may help bridge the conceptual divide between digital and traditional psychiatric assessment.

### Conclusions

This implementation suggests that wearable-derived sleep and activity data reporting is feasible in inpatient psychiatry, as demonstrated by a high and consistent rate of patient participation and technical report generation. This data reporting potentially offers clinically meaningful insights, particularly when patient and staff sleep reports conflict, as demonstrated by limited but consistent use among 1 clinician of 3 on the unit where the implementation took place, who served as an early adopter and contributor to the implementation (AGY). Additionally, reports were thought to be potentially useful by a convenience sample of 7 psychiatrists at the hospital who reviewed the reports but did not use them in clinical practice. Sustainable use and broader uptake are more likely with reliable, near-instantaneous data transfer; EMR integration; and shared implementation ownership across staff levels.

## Supplementary material

10.2196/88466 Multimedia Appendix 1 Narrative descriptions of phase 1 and phase 2.

10.2196/88466 Multimedia Appendix 2 AI transcript.

10.2196/88466 Checklist 1 iCHECK-DH checklist.

## Abbreviations

EMR: electronic medical record
HIPAA: Health Insurance Portability and Accountability Act
iCHECK-DH: Guidelines and Checklist for the Reporting on Digital Health Implementations
