# Supplementary material for: Clinical Implementation of Wearable-Derived Sleep and Activity Reporting for Inpatient Psychiatric Monitoring
Source: JMIR Form Res. 2026 Sep 21;10:e88466. doi: 10.2196/88466 (PMC13592238; doi:10.2196/88466)
Supplement: Supplementary file 1 [file formative-v10-e88466-s001.docx]

**Narrative Descriptions of Phase I and Phase II**

In phase one, a concerted effort was made to offer devices to all patients admitted to the unit and track patient participation. Devices were collected for data processing by a research assistant. Initially, reports were requested via QR code. Given low uptake of the QR request model, we elected to deliver weekly actigraphy reports to clinicians for all participating patients. We experimented with delivering key findings from the actigraphy reports in clinical rounds once a week—first by research assistant, then by briefing nurses to deliver key findings. Neither research assistant nor nursing presentation of reports gelled with the flow of rounds during this phase. After three months of experimentation, we scaled down our efforts on the unit and regrouped.

In phase two, we refined our process and explored challenges discovered in phase one, while keeping efforts and resources required of unit staff low. We returned to a request model, now completed by email. Reports were delivered by secure file transfer using Dropbox or email and report generation timetables were tracked throughout. Reports were fully automated, greatly reducing generation and turnaround time. During this phase, interviews were conducted with inpatient clinical staff to assess clinician views on the utility of sleep and activity information as well as to survey general impressions of our report prototype. We conducted semi-structured interviews with five unit mental health staff, four nurses, two social workers, and eight psychiatrists. Only one of the interviewed psychiatrists was directly involved in the initiative. Interviewing psychiatrists uninvolved in the initiative allowed us to assess psychiatrists’ first impressions of our updated report prototype. In review of the initiative, we interviewed the Medical Director and the Nursing Director. The Medical Director was one of three psychiatrists on the unit at the time of the initiative. By the time of interviews, one psychiatrist had left the hospital and the other had never requested a report but had given feedback on reports delivered in rounds—essentially that reports were not providing much new information in their view. General nursing sentiment for the project was captured through the project research assistant’s experience of the project, as well as the Nursing Director’s and Medical Director’s interviews.

Phase I

Phase one of the project spanned August–December of 2023. Nurses were asked to offer devices to patients at admission and track the number of patients approached, how many accepted, how many declined, and the reasons given for declination. Reports were initially requested through a survey form linked via QR code on flyers posted in the unit’s nursing station. Within 24 hours of a report request, a research assistant would swap out the patient’s device, download the data from the worn device, and upload the data to study servers. Then automated software would produce a graphical report integrating patient sleep data with electronic health record data. This report would be reviewed for errors by lab staff before being delivered to the requesting clinician via email, typically within 48 hours of request. While using this system, only six report requests were completed. The bulk of the requests came from the medical director acting as a project champion on the unit. We received no requests from nurses or social workers.

We next elected to produce weekly reports for all patients participating in the project. Under this system, all participant devices worn 3+ days were swapped out every Thursday. By end of day, all data were downloaded from the devices and uploaded to study servers. Automated in house software generated graphical reports for all uploaded data. We added a written summary to the report modeled after a radiology report. The written summaries were created in response to a sense that reports were not immediately comprehensible to new users and required some effort to understand and interpret. Written summaries of the reports were generated on Sundays by a member of the lab most familiar with the devices and data. Reports were reviewed on Mondays and presented during clinical rounds on Tuesdays by reading the Impressions section of the written report, which aimed to summarize the most useful data of the report in two sentences. Responses to this system were mixed, and the insertion of research staff into rounds was awkward, so we iterated again.

In attempts to further integrate this system into clinical practice, we next experimented with delivering reports to nurses, highlighting the key findings of the reports and asking nurses to present these findings to clinicians in rounds. During this iteration, improvements in report generation allowed for much of the written report to be automated; those pieces of the report still written by hand were completed on Sundays and reviewed on Mondays. On Tuesdays, a member of the research team would meet with nurses prior to rounds, review the impressions section with nurses and sit in on rounds to listen. While this iteration better integrated reports into clinical workflow, presentation of the reports still felt unnatural given the disconnect between the way nurses are used to talking about sleep and the percentile-driven language of the sleep reports. Additionally, reports did not seem to contribute greatly to rounds at this point. Here, we chose to conclude the first phase of the project to reflect and revise.

Phase II

At the conclusion of phase one, the research team decided to take a step back and reassess procedures and demand; we used phase two as a chance to refine aspects of the process and explore challenges recognized in phase one, while scaling down our effort and resources spent on the unit. During phase two, we elected to return to a request model, now completed by email request. Device administration was still performed by nurses, now at clinician request. During this phase, reports became fully automated, greatly reducing generation time. Reports were delivered via secure file transfer using Dropbox or by email and report generation timetables were tracked throughout this phase of the project.
