# Supplementary material for: Clinical Implementation of Wearable-Derived Sleep and Activity Reporting for Inpatient Psychiatric Monitoring
Source: JMIR Form Res. 2026 Sep 21;10:e88466. doi: 10.2196/88466 (PMC13592238; doi:10.2196/88466)
Supplement: Supplementary file 3 [file formative-v10-e88466-s003.docx]

**iCHECK-DH Checklist**

| **Section** | **Number** | **Section in Paper** |
| --- | --- | --- |
| **TITLE** | 1 | Title: Clinical Implementation of Wearable-Derived Sleep and Activity Reporting for Inpatient Monitoring |
| **ABSTRACT** | 2 | Abstract |
| **INTRODUCTION** | 3  4  5 | Context  Problem Statement  Similar Interventions |
| **METHODS** | 6  7  8  9  10  11  12  13  14 | Aim and Objectives  Blueprint Summary  Technical Design  Target  Data  Interoperability  Participating Entities  Budget Planning  Sustainability |
| **RESULTS / IMPLEMENTATION** | 15  16  17  18 | Coverage  Outcomes  Operational Outcomes  Clinical Uptake  Clinician Impressions from Prototype Review  Patient and Staff Experience  Lessons Learned  Guiding Principles for Design  Barriers and Facilitators  Success Factors and Sustainability  Unintended Consequences – Omitted. None to report |
| **DISCUSSION** | 19 | Discussion  Principal Findings  Limitations  Future Recommendations  Conclusion |
| **GENERAL** | 20 | Omitted. Not applicable to this paper |
